# Hydroxyethyl starch for volume expansion after subarachnoid haemorrhage and renal function: Results of a retrospective analysis

**DOI:** 10.1371/journal.pone.0192832

**Published:** 2018-02-15

**Authors:** Sven Bercker, Tanja Winkelmann, Thilo Busch, Sven Laudi, Dirk Lindner, Jürgen Meixensberger

**Affiliations:** 1 Department of Anaesthesiology and Intensive Care Medicine, University Hospital Leipzig, Leipzig, Germany; 2 Department of Neurosurgery, University Hospital Leipzig, Leipzig, Germany; Fraunhofer Research Institution of Marine Biotechnology, GERMANY

## Abstract

**Background:**

Hydroxyethyl starch (HES) was part of “triple-H” therapy for prophylaxis and therapy of vasospasm in patients with subarachnoid haemorrhage (SAH). The European Medicines Agency restricted the use of HES in 2013 due to an increase of renal failure in critically ill patients receiving HES compared to crystalloid fluids. The occurrence of renal insufficiency in patients with SAH due to HES is still uncertain. The purpose of our study was to evaluate whether there was an association with renal impairment in patients receiving HES after subarachnoid haemorrhage.

**Methods:**

Medical records of all non-traumatic SAH patients treated at the Departments of Anaesthesiology and Neurosurgery, University Hospital of Leipzig, Germany, between January 2009 and December 2014 were analysed. Patients received either HES 6% and/or 10% (HES group, n = 183) or exclusively crystalloids for fluid therapy (Crystalloid group, n = 93). Primary outcome was the incidence of acute kidney injury.

**Results:**

The study groups had similar characteristics except for initial SAPS scores, incidence of vasospasm and ICU length of stay. Patients receiving HES fulfilled significantly more often SIRS (systemic inflammatory response syndrome) criteria. 24.6% (45/183) of the patients in the HES group had acute kidney injury (KDIGO 1–3) at any time during their ICU stay compared to 26.9% (25/93) in the crystalloid group (p = 0.679). Only few patients needed renal replacement therapy with no significant difference between groups (Crystalloid group: 4.3%; HES group: 2.2%; p = 0.322). The incidence of vasospasm was increased in the HES group when compared to the crystalloid group (33.9% vs. 17.2%; p = 0.004).

**Conclusion:**

In the presented series of patients with non-traumatic SAH we found no significant association between HES therapy and the incidence of acute kidney injury. Treatment without HES did not worsen patient outcome.

## Introduction

About 43% of patients suffer from symptomatic vasospasm following subarachnoid haemorrhage (SAH) and 34% of these keep long-term disabilities [1]. Vasospasm leads to a substantial increase in morbidity and mortality after SAH. It has been estimated that vasospasm is causing 23% of deaths in patients with SAH [2]. Although the mechanism leading to narrowing of cerebral arteries after SAH has been the subject of extensive research, highly effective therapeutic or prophylactic treatments are still lacking. Efforts have been focused on improvement of regional blood flow by altering haemodynamics. Hence, “triple-H” therapy (hypervolaemia, hypertension, haemodilution) was established aiming at preventing cerebral infarction by improving perfusion [3]. To achieve effective and long-term plasma expansion, artificial colloids such as hydroxyethyl starch (HES) have been used in the past but the effects on regional blood flow and outcome remained controversial [4]. Since there is no substantial evidence supporting this concept instead of euvolaemia, current guidelines, however, do not recommend hypervolaemia anymore [5].

A few years ago concerns have been raised that the use of hydroxyethyl starch contributes to renal failure in critically ill patients. Starting with the VISEP trial evidence grew that HES solutions lead to an increased incidence of renal failure in septic patients [6–8]. Consecutively, regulatory authorities in the US and Europe limited the use of HES. However, its safety during elective surgery or in non-septic patients is still under debate. A recent meta-analysis of 22 randomized controlled trials comparing HES with different fluids (Gelatine, crystalloids or Albumin) in non-septic patients in intensive care units could not demonstrate a difference in the need for renal replacement therapy (RRT) [9]. On the other hand, impairment of renal function after HES administration could be demonstrated in non-septic elderly trauma patients [10].

The original concept of hypertension, hypervolaemia and haemodilution to prevent or treat vasospasm after SAH was modified over time and hypervolaemia has been abandoned in favour of avoiding hypovolaemia [11, 12]. Formerly, the use of hydroxyethyl starch was common for prophylaxis and treatment of hypovolaemia after subarachnoid haemorrhage. After initial concerns about nephrotoxic effects the use of HES 10% with a molecular weight of 200,000 Da has been restricted in our hospital since October 2011 and stopped in January 2012. However, low molecular HES 6% (130,000 Da) was in use until July 2013. After regulatory actions of the European Medicines Agency to restrict its application in critically ill patients and according to current guidelines [5], only crystalloids were used to maintain euvolaemia in SAH patients since 2014. Apart from this modification the treatment of SAH remained unchanged. This fact allowed comparing the incidence of renal failure in patients with different regimens of intravascular volume therapy. We hereby present a single-centre retrospective analysis of patients with SAH receiving either different types of HES or crystalloids.

## Methods

Patients were identified by selecting SAH as primary diagnosis in the hospital information system (SAP SE, Walldorf Germany). Clinical data of these patients were analysed retrospectively using the computer-based patient data management system (COPRA System GmbH, Berlin, Germany). The local ethics committee approved the study and waived the need for informed consent.

SAH patients admitted to our ICU before February 2012 received HES 10% continuously via infusion system to prevent hypovolaemia. The standard fluid dose of HES 10% was 1,000 ml/24h and was started immediately after surgical or endovascular therapy. HES 6% was administered additionally as repetitive bolus application to treat hypovolaemia at the discretion of the attending physician until July 2013. Target MAP was 65 mmHg (in absence of increased intracerebral pressure) and norepinephrine was added to the therapy if it was not achieved by fluid therapy alone.

Fluid status was monitored in every patient via fluid balance, mean arterial pressure, central venous pressure, clinical assessment and changes in blood chemistry. Only few patients received a PiCCO catheter during periods of severe shock to adjust volume and catecholamine therapy. Application of crystalloids aimed at avoiding hypovolaemia and at maintaining a well-adjusted fluid balance.

Baseline parameter as well as data on subarachnoid haemorrhage (severity, classification), associated complications (vasospasm), ICU scores (Simplified Acute Physiology Score—SAPS, Acute Physiology and Chronic Health Evaluation—APACHE II), days on mechanical ventilation, ICU length of stay (ICU-LOS), comorbidities, mortality and the Glasgow Outcome Score (GOS) were extracted [13, 14]. Systemic inflammatory response syndrome (SIRS) was diagnosed when at least two SIRS criteria were fulfilled as defined by Bone [15]. The severity of SAH was classified both by Hunt and Hess Score and by WFNS grade [16, 17]. Additionally the extent of bleeding in X-ray computed tomography (CT) scan was graded by Fisher scale [18]. All patients received routine transcranial Doppler ultrasound (TCD) for monitoring of vasospasm. Vasospasm was defined as critical increase of blood flow velocity (> 160 cm/sec) and/or angiographic vasoconstriction of the circle of Willis [19, 20].

To assess risk factors for renal failure independently from HES application, pre-existing renal impairment (defined by a creatinine clearance < 90 ml/min upon admission or defined by pre-existing history of chronic renal failure), episodes of sepsis and the application of nephrotoxic substances were analysed.

Acute kidney injury was determined and graded according to the definition of the Kidney Disease Improving Global Outcomes (KDIGO) Acute Kidney Injury Work Group [21]. Three severity stages reclassify the earlier used RIFLE criteria (KDIGO stage 1 = RIFLE risk for injury; KDIGO stage 2 = RIFLE injury; KDIGO stage 3 = RIFLE failure, or more severe) [22]. The stages are defined by certain increases in serum creatinine (stage 1: ≥0,3 mg /dl or 1.5–1.9 fold; stage 2: 2–2.9 fold; stage 3: ≥3 fold or ≥ 4 mg) and/or by a specified urine output (stage 1: < 0.5 ml/kg/h for 6–12 h; stage 2: < 0.5 ml/kg/h for ≥12 h; stage 3: < 0.3 ml/kg/h for ≥24 h or anuria for ≥12 h) [21]. The creatinine clearance was approximately calculated based on age, body weight, and serum creatinine as proposed by Cockcroft and Gault [23]. Additionally, any use of renal replacement therapy (RRT) was documented. To study the effect of HES on renal function the cumulative doses for HES 10% and HES 6% were calculated for every patient.

For statistical analysis all patients were divided into 2 groups, depending on whether they received HES or not (HES group, n = 183; Crystalloid group, n = 93). Statistical analysis was performed using SPSS version 24 (IBM Corp. Amonk, N.Y., U.S.A.). Normal distribution of numerical variables was investigated with the Kolmogorov-Smirnov test. Differences between normally distributed variables were analysed with Student’s t-test; in case of other distributions the Mann-Whitney U-test was used. Nominal variables were compared using either the chi-2 test or Fisher’s exact test if appropriate. Receiver-operating characteristics (ROC) analysis was used to investigate the relationship between the HES dose and the incidence of acute kidney injury in the subgroup of patients receiving HES. Hierarchical log-linear analysis with backward elimination was used to investigate associations between acute kidney injury, pre-existing renal dysfunction and HES application. In a post hoc analysis factors influencing the incidence of pre-existing renal dysfunction were investigated by logistic regression. Two-sided p values < 0.05 were considered statistically significant. Numerical data are presented as median with interquartile range.

## Results

Between January 2009 and December 2014 16,348 patients have been treated in the interdisciplinary surgical ICU at the University Hospital of Leipzig. A primary diagnosis of SAH was documented in 276 patients. Out of these, n = 183 received HES (HES group; n = 106 with HES 6% and/or HES 10%; n = 77 with only HES 6%) whereas n = 93 were treated only with crystalloids as volume therapy (Crystalloid group). There was only rare use of other colloids; until 2012 few selected patients received small amounts of gelatine infusions. HES 10% was used only in rare cases after January 2011 and the use of HES 6% declined in 2013.

There were no differences between groups with respect to age, weight, gender, or APACHE II at admission. In contrast, patients of the crystalloid group had a significant lower SAPS at admission (42 (25.5–75) vs. 49 (31–63); p<0.05) and a lower average SAPS during ICU stay (Table 1).

**Table 1:** Baseline characteristics.

	Crystalloid	HES	p value
Age [years]	55 (47;71)	53 (45;66)	0.237
Weight [kg]	75 (65;82.5)	70 (62;80)	0.862
Height [cm]	169 (165;178)	168 (165;175)	0.691
APACHE II [points]	14 (10;21)	15 (10;23)	0.342
SAPS day 0 [points]	42 (25.5;57)	49 (31;63)	0.009*
SAPS average [points]	30.4 (20.8;41.2)	32.9 (22.2;43.2)	0.026*
SAPS max [points]	47 (33;63)	53 (35.7;63)	0.063

Data are given as median and interquartile range (p25; p75).

*significant difference between crystalloid group and HES group.

APACHE II: acute physiology and chronic health evaluation score II; SAPS: simplified acute physiology and chronic health evaluation score.

No differences were detected in the severity of SAH graduated by Hunt & Hess Score, WFNS grade and Fisher scale as shown in Table 2. Patients in the HES group had a significantly increased ICU-LOS and a higher incidence of vasospasm, whereas there were no differences in GOS and hospital mortality. From the 38 deceased patients 31 were diagnosed with brain death, and therapy was withdrawn following the patients will or due to futility in 4 patients. Three patients died from multiple organ failure.

**Table 2:** Severity of subarachnoid haemorrhage, incidence of vasospasm and outcome data.

	Crystalloid	HES	p value
Hunt & Hess Score [1–5]	2 (2;4)	3 (2;4)	0.428
WFNS grade [1–4]	2 (2;4)	2(2;4)	0.37
Fisher grade [1–4]	4 (3;4)	4 (3;4)	0.139
Incidence of vasospasm (%)	16/93 (17.2%)	62/183 (33.9%)	0.004*
ICU length of stay [days]	13 (8;23,5)	19 (12;28)	0.001*
Glasgow Outcome Scale [1–5]	4 (2;5)	3(2;5)	0.335
In-hospital mortality (%)	16/93 (17.2%)	22/183 (12.0%)	0.344

Data are given as median and interquartile range (p25; p75).

* significant difference between crystalloid group and HES group;

WFNS scale: World Federation of Neurosurgical Societies scale.

Impaired renal function at admission was reported in 36.6% (Crystalloid group) vs. 27.3% (HES group) (p = 0.115, Table 3). 51.6% patients of the crystalloid group vs. 38.3% of the HES group had arterial hypertension and/or diabetes mellitus (aHT/DM) as additional risk factors for acute renal injury (p = 0.034).

**Table 3:** Additional risk factors and occurrence of acute kidney injury.

	Crystalloid (n = 93)	HES (n = 183)	p value
Impaired renal function at admission	34 (36.6%)	50 (27.3%)	0.115
Arterial hypertension and/or DM	48 (51.6%)	70 (38.3%)	0.034*
SIRS	65 (69.9%)	150 (82.0%)	0.022*
Acute kidney injury (KDIGO 1–3)	25 (26.9%)	45 (24.6%)	0.679
Severe acute kidney injury (KDIGO 2–3)	16 (17.2%)	15 (8.2%)	0.025*
Patients with RRT	4 (4.3%)	4 (2.2%)	0.322

Data are given as absolute numbers and percentage.

* significant difference between crystalloid group and HES group.

“Impaired renal function at admission” is defined by a reduced creatinine clearance at admission or a known history of pre-existing chronic renal failure.

“Arterial hypertension and/or DM” is defined by a known history of hypertension or diabetes mellitus prior to admission.

“SIRS” is defined by any episode of fulfilling SIRS criteria during ICU stay.

We found a significantly higher incidence of SIRS in patients receiving HES when compared to the crystalloid group (82.0% vs. 69.9%, p = 0.022, Table 3). There were no differences between the HES group and the crystalloid group with respect to procalcitonine and serum lactate values or in the number of days with abnormal white blood cell counts when only patients with SIRS were considered. Cumulative days with SIRS criteria were different in both groups (HES group: 8 (5–15) days; Crystalloid group 6 (2–12) days; p = 0.017). Only 3 patients died of multiple organ failure as a consequence of SIRS. Two out of them received crystalloids and developed acute kidney injury. The third patient was part of the HES group and did not develop acute kidney injury. N = 176 out of 276 patients (62.8%) had at least one period of antibiotic treatment during the study period.

In 25 out of 93 cases (26.9%), patients of the crystalloid group showed acute kidney injury (KDIGO 1–3) at any time whereas HES patients had acute kidney injury in 45/183 cases (24.6%) (p = 0.679). The need of renal replacement therapy was overall low with no significant difference between groups (4.3% in the crystalloid group vs. 2.2% in the HES group; p = 0.322) (Table 3). Duration of acute kidney injury in the HES group was significantly lower when compared to the crystalloid group (2 (1–4) days vs. 3 (2–5) days; p = 0.012). Acute kidney injury (KDIGO 1–3) without pre-existing renal dysfunction was diagnosed in 14/93 (15.1%) patients of the crystalloid group and in 25/183 (13.7%) patients of the HES group (p = 0.754). There was a reduced incidence of severe forms of acute kidney injury (KDIGO 2–3) in the HES group when compared to the crystalloid group (8.2% vs. 17.2%; p = 0.025).

Application of HES was generally started at the first ICU day. The total dose of HES was 11.6 (4.0–19.1) g/kg resulting from 7.7 (0–17) g/kg HES 10% and 1.5 (0.5–4.4) g/kg HES 6%. HES 10% was applied for 11 (8–14) days. Duration of application of HES 6% was 2 (1–5) days in patients receiving both HES preparations and 5 (2–10) days when it was used separately.

We found no differences with respect to the HES dosage between patients receiving HES with (n = 45) or without (n = 138) acute kidney injury (total dose: 9.4 (2.3–16.4) vs. 12.8 (4.9–19.7) g/kg, p = 0.154; HES 10%: 1.6 (0.0–14.4) vs. 10.1 (0.0–17.4) g/kg, p = 0.208; HES 6% 2.2 (0.8–4.0) vs.1.4 (0.4–4.9) g/kg, p = 0.282).

A subgroup analysis comparing patients who received HES 10% alone or in combination with HES 6% (n = 106) with patients receiving only HES 6% (n = 77) revealed no differences between both groups with respect to incidence of acute kidney injury (23.6% vs. 26.0%, p = 0.711) and of its severe forms with KDIGO stage > 1 (7.5% vs. 9.1%, p = 0.707). All patients with severe acute kidney injury were classified as KDIGO 2 with exception of one patient with KDIGO 3 in each subgroup.

There was a clear trend for a reduced total HES dose in patients with pre-existing renal dysfunction when compared to patients without this diagnosis (7.4 (1.7–17.9) g/kg vs. 12.6 (5.2–19.4) g/kg; p = 0.052). In addition, we divided the patients receiving HES in two subgroups with (n = 45) and without (n = 138) acute kidney injury. There were no differences with respect to the HES dosage (total dose: 9.4 (2.3–16.4) vs. 12.8 (4.9–19.7) g/kg, p = 0.154; HES 10%: 1.6 (0.0–14.4) vs. 10.1 (0.0–17.4) g/kg, p = 0.208; HES 6% 2.2 (0.8–4.0) vs.1.4 (0.4–4.9) g/kg, p = 0.282). Neither the HES 10% nor the HES 6% dosage did predict the occurrence of acute kidney injury (ROC analysis with values for the area under the curve (AUC) of 0.440 (p = 0.226, HES 10%) and of 0.447 (p = 0.283, HES 6%), respectively).

A three factorial hierarchical log-linear analysis resulted in a statistical model demonstrating only an association between acute kidney injury and pre-existing renal dysfunction on admission, which was independent of the HES application (goodness of fit likelihood ratio chi-square 3.561, p = 0.313 vs. a starting model containing all possible associations between the three parameters). Expansion of the log-linear model by the inclusion of aHT/DM as forth factor confirmed the results of the three factorial model. In addition, there was an association between pre-existing renal dysfunction and aHT/DM, but no association between acute kidney injury and aHT/DM. A further association between aHT/DM and HES application occurred due to the significant difference of aHT/DM between the crystalloid and the HES group (goodness of fit likelihood ratio chi-square 7.183, p = 0.517 vs. a starting model containing all possible associations between the four parameters).

## Discussion

We hereby present a study on renal failure in patients with non-traumatic subarachnoid haemorrhage treated with hydroxyethyl starch. During the last decades clinically available and used intravenous fluids changed, therefore we compared different groups of patients with SAH treated with different regimens of intravenous fluid administration. In contrast to recently published studies in critically ill patients we could not demonstrate an association between HES application or its cumulative doses and renal dysfunction or the necessity to apply renal replacement therapy [6–8]. The incidence of acute kidney injury in our patients depended on the pre-existing renal dysfunction, but not on the HES application. It was reversible in most cases as indicated by the low frequency of renal replacement therapy. The higher duration of acute kidney injury in the crystalloid group when compared to the HES group might be explained by the higher rate of severe forms of acute kidney injury with KDIGO state 2 and 3 in the crystalloid group.

The higher incidence of pre-existing renal dysfunction in the crystalloid group compared to the HES group remains to be explained. In an univariate post hoc analysis patients with pre-existing renal dysfunction (n = 84) were older (70 (59, 77) vs. 49 (43, 57) y; p>0.001), were smaller (165 (160, 173) vs. 170 (165, 175) cm; p = 0.011), had a higher APACHE II score (17 (12, 24) vs. 13 (9, 21); p = 0.002), had a higher SAPS score on admission (52 (40, 62) vs. 44 (24, 59); p = 0.007), and had a higher frequency of arterial hypertension and/or diabetes mellitus (65.5% vs. 32.8%; p<0.001) when compared to patients without pre-existing renal dysfunction (n = 192). Multivariate logistic regression revealed only age as an independent predictor for pre-existing renal failure (OR 1.22 per year (95% confidence interval 1.089–1.155); p<0.001). Non-significant multivariate p values resulted together with aHT/DM (p = 0.684) for body height (p = 0.586), APACHE II score (p = 0.906), and SAPS score (p = 0.453), excluding these parameters as major determinants for pre-existing renal dysfunction. Importantly, age was not a normally distributed variable (Kolmogorov Smirnov test with Lilliefors correction resulted in p = 0.042 and p = 0.048 for crystalloid group and HES group, respectively). This implicates that the incidence of older patients might have been different between the groups. Correspondingly, we found that the frequency of patients older than 75 years was different (Crystalloid group: 18/93 (19.4%); HES group 17/183 (9.3%); p = 0.018). This age group (>75 years) was simultaneously more frequent in patients with pre-existing renal dysfunction when compared to patients without this diagnosis (29/84 (34.5%) vs. 6/192 (3.1%); p<0.001). Taken together, different fractions of patients with age greater 75 years might have contributed to the unequal incidence of pre-existing renal dysfunction in the HES and in the crystalloid group.

The discussion on using colloids as a volume expander during surgery, in emergency medicine and critical care lasts since decades and is still ongoing. The indication for artificial colloids still remains unclear and the most recent Cochrane analysis on this issue concludes that there is no evidence to the use of colloids in critically ill patients [24]. Furthermore, the use of HES in septic patients has been shown to impair renal function or even increase mortality [25]. However, data on non-septic patients are rather inconsistent. Several randomized single centre trials studying the use of HES during hip arthroplasty [26], or radical prostatectomy demonstrated its perioperative safety [27]. A meta-analysis of the perioperative use of HES in surgical patients could not draw sound conclusions since the data were insufficient [28]. A further analysis of studies with a variety of indications showed relevant risks only for septic patients while demonstrating possible protective effects in trauma patients [29].

No clear evidence exists on the use of either tetrastarch or pentastarch in patients with SAH, although these patients are generally treated in the ICU, are often critically ill, have a high incidence of septic complications, and should be therefore at high risk for renal impairment following HES infusions [30, 31]. The incidence of acute kidney injury in patients with SAH is more than 10% [32]. Additionally, it has been demonstrated that acute kidney injury is associated with a poor outcome [32, 33]. Potential risk factors for acute kidney injury are administration of large amounts of contrast dye, sepsis, shock, and the application of nephrotoxic substances, which are common in patients with SAH. Kunze et al. retrospectively described an increase of creatinine with peak values on day 3 in 107 SAH patients receiving HES 6% (130/0.4) as a standard treatment and reported no patient requiring RRT. However, in contrast to our study Kunze et al. included no comparison group [34].

Even though SAH is known to be associated with a high incidence of hospital acquired infections [31], the fact that 62.8% of our patients received antibiotics might indicate that a subset of patients might have been treated without distinct indication. However, taking into account that the incidence of SIRS was higher in the HES group, SIRS/sepsis seemed not to be a relevant co-factor for the development of renal impairment in this study.

Even if it was not the main goal of this study, it is remarkable that the HES group presented with a significantly higher rate of vasospasm. The fact that there were no significant differences in GOS and mortality between both groups indicates that the additional cases with vasospasm were less severe. However, retrospective diagnosis was not standardized and available data were of uncertain quality. Therefore, results must be interpreted with caution. Nevertheless, the lack of a beneficial effect of the HES application on the incidence of vasospasm provides arguments against the use of HES in SAH patients in spite of a missing significant impact of HES on the incidence of acute kidney injury. This is in line with the current practise to omit HES in SAH patients.

Despite the above-described risk factors and conflicting results from other studies in unselected critically ill patients, we could not demonstrate an association between acute kidney injury and the use of HES.

We only can hypothesize why the use of HES was not associated with the development of acute kidney injury in our patients:

- In our study patients presented with a rather low rate of severe multiple organ failure (MOF). In SAH mortality is rather determined by the cerebral insult then by MOF even if complications are frequent. Therefore, the influence of co-factors like HES administration might have been masked by cerebral complications.
- Even if acute kidney injury was frequent, most patients suffered from an only temporary increase in creatinine values or oliguria. As renal failure caused by the underlying disease and co-factors as described above is less severe than in patients with severe sepsis or septic shock, the additional effect of HES on renal function might have been of minor importance.
- Even if hypervolaemia has been abandoned for years, euvolaemia or at least the avoidance of hypovolaemia is an important goal of haemodynamic therapy in patients with SAH. The strict avoidance of hypovolaemia might have contributed to the low rate of severe renal failure and patients requiring RRT. Protective effects of this regimen on acute kidney injury might have counterbalanced toxic effects of HES.

The most important limitation of our study is its retrospective character and its limited sample size. It has to be noted that the non-significant (p = 0.679) difference in the incidence of acute kidney injury (KDIGO 1–3) of 26.9% in the crystalloid group and of 24.9% in the HES group does not exclude the existence of an association between HES application and acute kidney injury. This is reflected by the relative risk (RR) for acute kidney injury due to HES application of 0.915 together with its 95% confidence interval (CI) ranging from 0.599 to 1.396. Similar data in critically ill patients communicated from Guidet et al. (n = 196 with sepsis; RR 0.92, 95% CI 0.55–1.53) and from Magder et al. (n = 236 after cardiac surgery; RR 0.9, 95% CI 0.54–1.51) were included in a current Cochrane meta-analysis with 8,769 patients which revealed a RR for acute kidney injury corresponding to KDIGO 1–3 (or RIFLE risk or worse) with a value of 0.95 (95% CI 0.91–0.99) [35–37]. The absolute difference in the incidence of acute kidney injury (KDIGO 1–3) between our HES and crystalloid groups of 2.5% has a 95% confidence interval ranging from a -13.3% reduction to an increase of 8.7% caused by HES. Our study was not powered to detect values within this interval as significant. A retrospective power calculation based on the incidence of acute kidney injury (KDIGO 1–3) in our crystalloid group revealed that our sample size of 276 SAH patients would identify only a difference of 14.7 percentage points with a two-sided p<0.05 and 80% statistical power in a controlled study; this calculation was performed using a formula derived by Fleiss et al. [38]. We found a significantly decreased incidence of severe acute kidney injury (KDIGO 2–3) in the HES group, but this was the result of a post hoc analysis. Despite these shortcomings our investigation is to our best knowledge currently the only available clinical study comparing patients receiving HES with those who did not following subarachnoid haemorrhage. Second, there was an unequal distribution of baseline parameters between groups. The higher SAPS scores as well as the increased ICU-LOS indicate that patients who received HES presented more severely ill. Despite these additional risk factors for renal impairment there were no differences in renal failure or RRT and a non-significant lower mortality in SAH patients receiving HES. Therefore, these limitations do not weaken our main finding.

## Conclusion

In summary, we could not demonstrate a statistically significant association between the application of HES and the incidence of acute kidney injury in patients with SAH. Additionally, as the incidence of vasospasm was higher in the HES group, our data suggest that omitting HES was not associated with harm in SAH patients. These results provide arguments that termination of the HES application in our SAH patients was justified. In the light of the retrospective character of the study results should be interpreted with caution.

## Supporting information

S1 Table Available dataset. (XLS)
